# The 10,000-year biocultural history of fallow deer and its implications for conservation policy

**DOI:** 10.1073/pnas.2310051121

**Published:** 2024-02-12

**Authors:** Karis H. Baker, Holly Miller, Sean Doherty, Howard W. I. Gray, Julie Daujat, Canan Çakırlar, Nikolai Spassov, Katerina Trantalidou, Richard Madgwick, Angela L. Lamb, Carly Ameen, Levent Atici, Polydora Baker, Fiona Beglane, Helene Benkert, Robin Bendrey, Annelise Binois-Roman, Ruth F. Carden, Antonio Curci, Bea De Cupere, Cleia Detry, Erika Gál, Chloé Genies, Günther K. Kunst, Robert Liddiard, Rebecca Nicholson, Sophia Perdikaris, Joris Peters, Fabienne Pigière, Aleksander G. Pluskowski, Peta Sadler, Sandra Sicard, Lena Strid, Jack Sudds, Robert Symmons, Katie Tardio, Alejandro Valenzuela, Monique van Veen, Sonja Vuković, Jaco Weinstock, Barbara Wilkens, Roger J. A. Wilson, Jane A. Evans, A. Rus Hoelzel, Naomi Sykes

**Affiliations:** ^a^Department of Biosciences, Durham University, Durham DH1 3LE, United Kingdom; ^b^Department of Classics and Archaeology, University of Nottingham, Nottingham NG7 2RD, United Kingdom; ^c^Department of Archaeology and History, University of Exeter, Exeter EX4 4QE, United Kingdom; ^d^Groningen Institute of Archaeology, University of Groningen, Groningen 9712 ER, The Netherlands; ^e^Department of Paleontology, National Museum of Natural History, Bulgarian Academy of Sciences, Sofia 1000, Bulgaria; ^f^Ephorate for Palaeoanthropology-Speleology, Hellenic Ministry of Culture, Athens 106 82, Greece; ^g^School of History, Archaeology and Religion, Cardiff University, Cardiff CF10 3EU, United Kingdom; ^h^National Environmental Isotope Facility, British Geological Survey, Nottingham NG12 5GG, United Kingdom; ^i^Department of Anthropology, University of Nevada, Las Vegas, NV 89154; ^j^Historic England, Portsmouth PO4 9LD, United Kingdom; ^k^Centre for Environmental Research Innovation and Sustainability, Atlantic Technological University, Sligo F91 YW50, Ireland; ^l^School of History, Classics and Archaeology, University of Edinburgh, Edinburgh EH8 9AG, United Kingdom; ^m^School of Art History and Archaeology, University of Paris 1 Panthéon-Sorbonne, Paris 75006, France; ^n^School of Archaeology, University College Dublin, Dublin D04 V1W8, Ireland; ^o^Department of History and Cultures, University of Bologna, Bologna 40124, Italy; ^p^Operational Directorate Earth and History of Life, Royal Belgian Institute of Natural Sciences, Brussels 1000, Belgium; ^q^Center of Archaeology of the University of Lisbon, Department of History, School of Arts and Humanities of the University of Lisbon, Alameda da Universidade, Lisboa 1600-214, Portugal; ^r^Institute of Archaeology, HUN-REN Research Centre for the Humanities, Budapest 1097, Hungary; ^s^Bureau d’études, Éveha, Saint-Avertin, Tour 37550, France; ^t^Vienna Institute for Archaeological Science, Research Network Human Evolution and Archaeological Sciences, University of Vienna, Vienna 1090, Austria; ^u^School of History, University of East Anglia, Norwich Research Park, Norwich NR4 7TJX, United Kingdom; ^v^Oxford Archaeology Ltd., Osney Mead, Oxford OX2 0ES, United Kingdom; ^w^School of Global Integrative Studies, University of Nebraska-Lincoln, Lincoln, NE 68588; ^x^Institute of Palaeoanatomy, Domestication Research and the History of Veterinary Medicine, Department of Veterinary Sciences, Ludwig Maximilian University of Munich, Munich 80539, Germany; ^y^Bavarian Natural History Collections, State Collection of Palaeoanatomy Munich, Munich 80333, Germany; ^z^Department of Geography, Royal Holloway, University of London, Egham TW20 0EX, United Kingdom; ^aa^Department of Archaeology, University of Reading, Reading RG6 6AX, United Kingdom; ^bb^Independent Researcher, Buckinghamshire, Greater Missenden HP16 0LF, United Kingdom; ^cc^Département de la Charente, Angouleme Cedex 9 1616917, France; ^dd^Department of Archaeology and Ancient History, Lund University, Lund 223 62, Sweden; ^ee^Fishbourne Roman Palace, Chichester PO19 3QR, United Kingdom; ^ff^Department of Classics and Ancient Mediterranean Studies, Bucknell University, Lewisburg, PA 17837; ^gg^Mediterranean Institute for Advanced Studies, Ecology and Evolution, Miquel Marquès Street, Esporles, Illes Balears 2107190, Spain; ^hh^Department of Archaeology, Municipality of The Hague, Den Haag 2500 DP, The Netherlands; ^ii^Laboratory for Bioarchaeology, Archaeology Department, University of Belgrade, Belgrade 11000, Serbia; ^jj^Department of Archaeology, University of Southampton, School of Humanities, Southampton SO171BF, United Kingdom; ^kk^Independent Researcher, Alghero, Vancouver 07041, Italy; ^ll^Department of Ancient Mediterranean and Near Eastern Studies, V6T 1Z1, Canada

**Keywords:** fallow deer, translocations, extinctions, zooarchaeology, biomolecules

## Abstract

Over the last 10,000 y, humans have manipulated fallow deer populations with varying outcomes. Persian fallow deer (*Dama mesopotamica*) are now endangered. European fallow deer (*Dama dama*) are globally widespread and are simultaneously considered wild, domestic, endangered, invasive and are even the national animal of Barbuda and Antigua. Despite their close association with people, there is no consensus regarding their natural ranges or the timing and circumstances of their human-mediated translocations and extirpations. Our mitochondrial analyses of modern and archaeological specimens revealed two distinct clades of European fallow deer present in Anatolia and the Balkans. Zooarchaeological evidence suggests these regions were their sole glacial refugia. By combining biomolecular analyses with archaeological and textual evidence, we chart the declining distribution of Persian fallow deer and demonstrate that humans repeatedly translocated European fallow deer, sourced from the most geographically distant populations. Deer taken to Neolithic Chios and Rhodes derived not from nearby Anatolia, but from the Balkans. Though fallow deer were translocated throughout the Mediterranean as part of their association with the Greco-Roman goddesses Artemis and Diana, deer taken to Roman Mallorca were not locally available *Dama dama*, but *Dama mesopotamica*. Romans also initially introduced fallow deer to Northern Europe but the species became extinct and was reintroduced in the medieval period, this time from Anatolia. European colonial powers then transported deer populations across the globe. The biocultural histories of fallow deer challenge preconceptions about the divisions between wild and domestic species and provide information that should underpin modern management strategies.

## Significance

Persian and European fallow deer formed part of the Levantine Neolithic Package were worshiped as Greco-Roman deities and, over millennia, were the subject of repeated human-mediated translocations and extirpations. By integrating biomolecular datasets with archaeological and textual evidence, we reveal these species’ biocultural histories—spanning their Glacial refugia to their global spread as symbols of colonial power. The deep histories of fallow deer highlight the problems inherent in dichotomous categorizations of all species as either wild or domestic. In addition, they complicate existing wildlife conservation strategies and offer alternative perspectives on the management of translocated animals.

There are two recognized species of fallow deer: the Persian (*Dama mesopotamica*) and the European (*Dama dama*). The Persian fallow deer was once widespread across Southwest Asia and the eastern Mediterranean, but following a severe population decline, the species is currently considered Endangered by the International Union for the Conservation of Nature (IUCN) (1). Conversely, the European fallow deer, native to the eastern Mediterranean, is classified as Least Concern due to their human-mediated translocation and establishment across Eurasia, Africa, America and Oceania (2, 3). Despite their large population size and broad distribution, their genetic diversity is very low, suggesting conservation vulnerability (4). The herd of European fallow deer at Güllük Daği-Termessos National Park (Turkey) is considered the last native wild population and, as such, has a protected status (5). Conservation measures extend to the *Dama* population on the nearby island of Rhodes, which is protected by Greek law (6).

The Rhodes *Dama* are thought to descend from a population of European fallow deer introduced ~7,000 y ago (6–8). Early farmers of the 6th–5th millennium BCE also established populations of European fallow deer on the islands of Lemnos, Lesvos, Chios, and Crete (9–11), whereas Persian fallow deer were transported to Cyprus ~10,000 y ago (12).

Both species were as heavily influenced by people as other taxa classically associated with the Neolithic Package including cattle, sheep, goats, and pigs. In addition, modern European fallow deer are farmed in their millions (13) and exhibit coat color variations indicative of selective breeding (14). Despite these characteristics, fallow deer are rarely included in large-scale reviews of domestic animals (12, 15). European fallow deer have been equally overlooked by conservation scientists, for whom the species’ domestic legacy has meant they are often considered an introduced alien or invasive threat and thus undeserving of protection (16–18).

As neither an accepted domesticated nor a “pristine” wild species, both Persian and European fallow deer have been under-researched relative to other cervids such as reindeer (*Rangifer tarandus*) and red deer (*Cervus elaphus*) which have been the subject of numerous studies concerning their ancient range and management (19–23). By contrast, there is no consensus regarding the European fallow deer’s glacial refugia or natural post-glacial distribution. While some have suggested a single refugium in Anatolia (2), others have claimed multiple refugia across Anatolia, the southern Balkans, Italy, Sicily, and Iberia (4, 24, 25). The timing and circumstances of the fallow deer’s anthropogenic translocations are equally obscure, although numerous human cultures have been held responsible including early Neolithic farmers, Phoenicians, Romans, Normans, and early modern imperialists (26).

Attempts to answer questions about the fallow deer’s history have relied largely on genetic studies of modern animals (4, 7, 8, 27). However, modern DNA has limited retrodictive power, especially when applied to species whose distributions have been heavily modified by humans (15, 28). Recent aDNA studies of fallow deer have demonstrated the necessity of a joined-up ancient-modern genetics approach (29–33), exemplified by Baker et al.’s (34) time-calibrated genetic analyses of fallow deer evolution in Europe from the last glacial period. There is also a need to integrate genetic analyses with other sources of biomolecular data, such as isotope studies, and rich empirical records from across the Humanities and Social Sciences, which together can be used to evidence the long-term management and cultural value of fallow deer.

Here, in order to characterize the glacial range of Persian and European fallow deer, we combined zooarchaeological and biomolecular analysis of ancient and modern *Dama* remains. To increase the power of our results, we integrated them with evidence from archaeology, historical sources, and iconography and show how ancient humans have shaped the modern-day distributions and management strategies of these two species. As such, they represent cultural heritage and arguably deserve protection by the United Nations Educational Scientific and Cultural Organization (UNESCO) as much as from wildlife conservation bodies such as the IUCN.

## Results and Discussion

We analyzed 635 osteological samples purported to derive from fallow deer using at least one method (Dataset S1) and generated genetic sequences from 228 ancient samples.

**Fig. 1.**
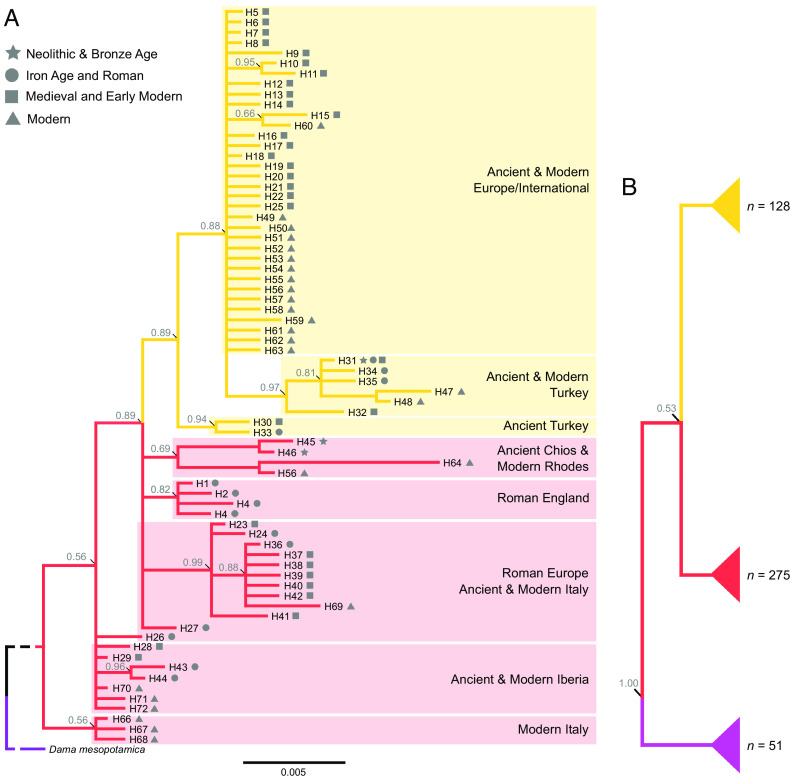
Phylogenetic trees depicting the relationships between mitochondrial haplotypes derived from ancient and modern European and Persian fallow deer. The tree in Panel (*A*), rooted with Persian fallow deer and based upon 532 basepairs, shows a distinctive well-supported (0.89 posterior probability) monophyletic clade of European fallow deer (depicted in yellow) and separate lineages of fallow deer associated with a population originally present in the Balkans (depicted in red). Individuals from both these populations (as well as Persian fallow deer) have been transported beyond their native ranges by people at different times (Fig. 2). The collapsed tree in Panel (*B*) is rooted to *Cervus elaphus* and based upon 128 basepairs (see *SI Appendix*, Fig. S1 for detailed tree). It shows how European fallow deer (yellow and red) can be differentiated from the well supported (1.0 posterior probability) clade of Persian fallow deer (purple).

For the European fallow deer, 181 sequences from archaeological samples (35) were combined with those for 222 modern individuals (4, 36). A Bayesian phylogeny constructed from the complete alignment (Fig. 1*A*) revealed a well-supported (0.89 posterior probability) monophyletic clade consisting of modern and ancient European fallow deer from Northern Europe and Anatolia (depicted in yellow on Fig. 1 *A* and *B*).

A second clade is made up of ancient and modern fallow deer derived from southern and western European sites and Roman England (depicted in red–Fig. 1 *A* and *B*). Both clades are distinct from the mitochondrial lineage derived from a single modern Persian fallow deer at the base of the phylogeny.

A shorter sequence was available (128 bp) that showed 18 fixed differences between European and Persian fallow deer, allowing *D. mesopotamica* to be identified from archaeological remains (depicted in purple in Fig. 1*B* and *SI Appendix*, Fig. S1)

**Fig. 2.**
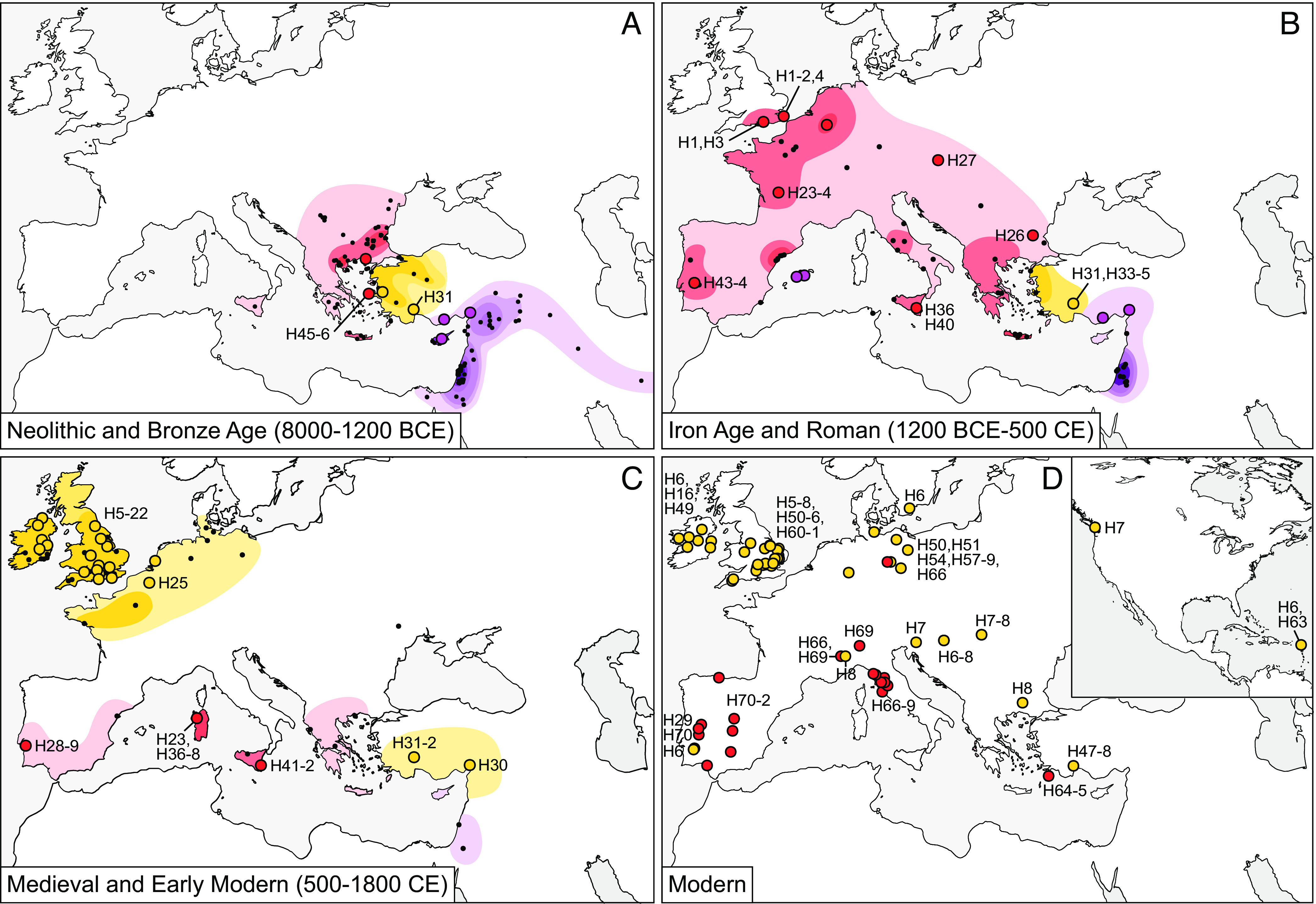
Maps showing the location and density of European and Persian fallow deer remains in zooarchaeological assemblages (*A*–*C*, data from Dataset S2) and modern samples (*D*, data from Datasets S3 and S4) colored according to genetic results (Fig. 1 and Dataset S1).

**Fig. 3.**
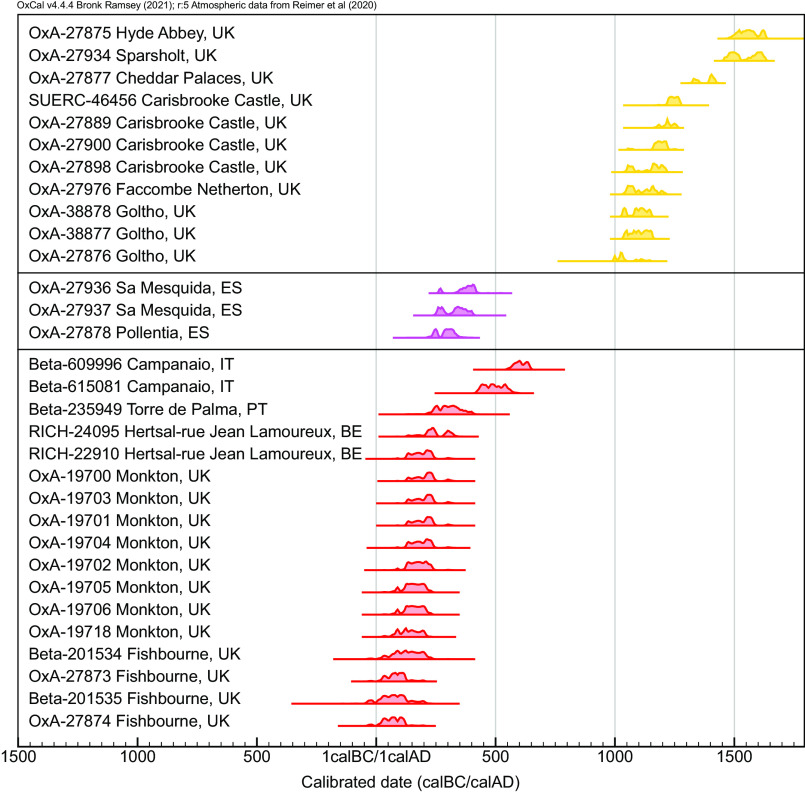
Calibrated radiocarbon dates of fallow deer, color-coded by genetic results (Fig. 1).

**Fig. 4.**
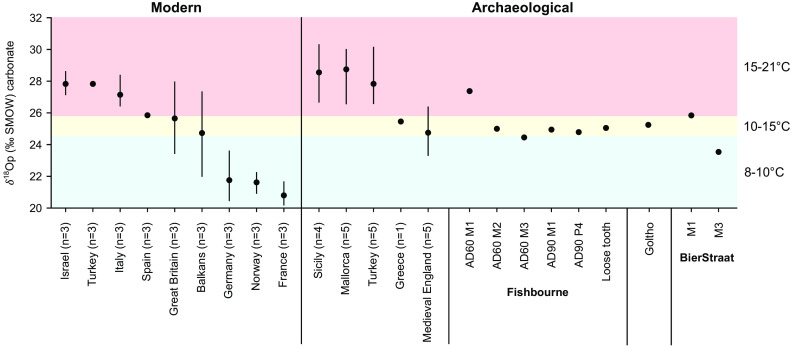
Range and mean of oxygen isotope data for modern fallow deer (37) compared with ancient specimens. The graph is colored by average temperatures for the regions from which the modern specimens derive. Those from ancient Turkey, Greece, Sicily, Mallorca, and medieval England are consistent with animals that lived and died in those regions. By contrast, the AD60 mandible from Fishbourne Roman Palace has a value for its first molar (M1, which develops <4 mo) that is more in line with those from Turkey, Israel, the Balkans, and Italy. The values for the same deer’s second molar (M2, develops 8 to 9 mo) and third molar (M3, develops <18 mo), along with those for the other Fishbourne fallow deer, align with those from modern and medieval England. The specimen from Goltho is consistent with UK values. The specimen from post-medieval Bierstraat-The Hague, the Netherlands, has an M1 value consistent with England but its M3 value is more suggestive of continental northern Europe.

The zooarchaeological representation data (Dataset S2) and genetic results are summarized in Fig. 2 *A*–*D*, which also incorporates the radiocarbon dating evidence (Fig. 3). Multi-element isotope data were generated from 418 specimens (Dataset S1), with results presented in Fig. 4 and *SI Appendix,* Figs. S6 and S7.

### Refugia and Native Range.

Our genetic analysis demonstrated that 38 specimens originally identified morphologically as *D. dama* were actually *D. mesopotamica* (*SI Appendix*, Fig. S1). Their presence at the Bronze Age/early Iron Age sites of Kinet Höyük and Kilise Tepe, Anatolia (Fig. 2 *A* and *B*) pushes the ancient distribution of Persian fallow deer further west than previously proposed (12).

For the European fallow deer, our data suggest their glacial refugium was restricted entirely to the eastern Mediterranean and there is no zooarchaeological evidence to suggest the existence of autochthonous Holocene fallow deer populations in Iberia or Italy. Within Anatolia, the *D. dama* population demonstrates continuity through time: Neolithic, Roman, and medieval deer share haplotypes (H31) and are closely related to the modern population at Güllük Daği-Termessos National Park (H47, H48). The modern deer population on Rhodes is genetically distinct from Anatolian deer [a result that corroborates previous studies (7, 8)] and appears more closely related to populations from the Balkans, Italy, and Iberia.

The phylogenetic split between the two populations of European fallow deer (Fig. 1) is consistent with the frequently observed phylogeographic divide found in numerous species with populations that span the Bosporus (38–40). When combined with the zooarchaeological data (Fig. 2*A*), this result supports the suggestion of a second glacial refugium in the southern and central Balkans (25, 41). Large quantities of fallow deer remains have been recovered from Neolithic and Bronze Age sites in Bulgaria which demonstrate their early presence in this region. Intriguingly, their remains exhibit morphologies distinct from the Anatolian fallow deer (*SI Appendix*, Figs. S3 and S4) which may be the result of underlying genetic, not environmental differences (25, 41, 42). Despite the phenotypic distinction, stable and radiogenic isotope data show no difference in fallow deer diets between these regions (*SI Appendix*, Fig. S6 and S7) (43).

### Translocations as Proxies for the Movement of People and Ideologies.

Both species of fallow deer were translocated during the Neolithic/Bronze Age (Fig. 2*A*). We partially sequenced one Persian fallow deer specimen (PT608) from the Bronze Age site of Politiko-Troullia, Cyprus, and two Late Neolithic/Early Bronze Age European fallow deer samples from Ayio Galas Cave, Chios (CH680 and CH681). The Chios samples possessed unique haplotypes (H45 and H46) that are most closely related to individuals from modern Rhodes (H64 and H65). This result supports Masseti et al.’s (7, 8) proposal that the modern Rhodes deer population descends from a Neolithic introduction.

The Neolithic Chios and modern Rhodes deer are more closely related to (and likely descend from) the Balkan rather than Anatolia population. This may seem counter-intuitive, especially given that Rhodes is only 11 miles from mainland Turkey and Chios is <3 miles. However, animal translocations frequently result from factors other than geographic proximity, including attitudes to the natural world, religious ideologies and culture-contacts, issues to which we now turn.

#### Early domestication.

The transfer of animals beyond their natural range has been equated with a closening of human–animal relationships and associated with the process of domestication (44).

According to Masseti (45) and Vigne et al. (12), island *Dama* populations were established specifically for hunting but textual and iconographic evidence from the Bronze Age indicate a more complex relationship between people and fallow deer. For instance, Linear B texts (the earliest form of Greek) list different kinds of fallow deer: those that are wild, those that are tame, and those used in games or for sacrifices, while fresco fragments from Aghia Triadha, Crete, depict a woman leading two fallow deer to a sacrificial altar (11). Similar evidence exists across Anatolia and Egypt (46) and given that the Latin name *Dama* derives from the Persian word for tame or pet (47), there is a compelling case that fallow deer were initially no different in their relationships with humans than other animals that make up the canonical suite of domesticates.

#### Religion.

Many cultures equate geographical distance with supernatural distance perceiving that the further something has traveled, the greater its prestige and power (48). In this way, introduced animals have frequently been viewed as gods (49, 50).

Fallow deer were certainly associated with both the goddess Artemis and her Roman incarnation, Diana (11, 46, 47, 51). There is debate about the geographical genesis of the Artemis myth but the possibility she originated in the Balkans is given credence by the density of both fallow deer remains (Fig. 2*A*) and Artemis-related paraphernalia, such as fallow deer-shaped religious drinking vessels that have been recovered from the region (52). In Late Minoan Crete (c.1550–1100 BCE), Linear B texts mention not only fallow deer but also provide the earliest reference to Artemis (10, 53).

Historical studies suggest that the Artemis cult was taken to Sicily by early Greek settlers (54) and statuettes of the goddess have been recovered from the Bronze Age site of Morgantina, together with a shed fallow deer antler (55). This skeletal element could have been transported as an object in its own right [as was the case for other *Dama* body parts recovered from a Phoenician ship-wreck off the coast of Sicily (43, 56)] rather than deriving from an animal that lived on the island. At Morgantina, a small number of post-cranial bones have been tentatively identified as *Dama* (55). Our metrical analysis shows that their size is more consistent with red deer from the island (*SI Appendix*, Fig. S5) though we were unable to confirm their identification genetically.

#### Roman Empire.

The Roman period witnessed a major expansion in fallow deer distribution (Fig. 2*B*). This was in part due to their connection with the goddess Diana and also linked to the parks and menageries that became increasingly fashionable throughout the Roman Empire.

The earliest evidence for the presence of fallow deer beyond the Mediterranean comes from the highly “Romanised” palatial site of Fishbourne (southern England) that was constructed shortly after the Roman invasion of Britain in 43 CE. Here, *Dama* remains have been directly dated to the 1st century CE (Fig. 3). Multi-element isotope analysis revealed a first-generation import that likely traveled from the Mediterranean in the first few months of its life (Fig. 4). Our evidence also shows that other fallow deer were born and raised at Fishbourne (57) and managed in diverse ways (58).

By the fourth-century CE, fallow deer were established in Britain more broadly, and specimens from Belgium (59) and Portugal (60) have been direct-dated to this period (Figs. 2*B* and 3). The earliest secure evidence for fallow deer on Sicily dates to the 5th century CE (Fig. 3 and *SI Appendix*, Fig. S3) and isotope analysis indicates these animals were born and raised on the island (Fig. 4). Genetically, Sicilian deer are consistent with the western Mediterranean (Balkan) clade. They share a haplotype (H36) with animals from Sardinia, where populations were established in the medieval period (61, 62). The Sardinian deer also share haplotypes (H23) with deer from Roman France.

The European fallow deer established in Iberia and Italy both appear to be the progenitors of the modern populations in those regions. For instance, one haplotype (H29) is observed in both medieval and modern Portugal, and is closely related to Roman haplotypes (H43 and H44). Similarly, haplotypes found in ancient Italy (H36) are closely related to those of modern deer (H69). These modern populations are therefore legacies of the Roman Empire and should be treated as living cultural heritage (Fig. 2 *B* and *D*).

Large numbers of *Dama* remains have been recovered on Mallorca in contexts dating from the third to the fifth century AD (31). Surprisingly, they were genetically determined to be Persian and not European fallow deer (*SI Appendix*, Fig. S1), which has implications for understanding Roman and early medieval trade routes. For instance, it is possible they arrived via north Africa where there are iconographic representations of fallow deer. Recently, zooarchaeological evidence for fallow deer has been discovered in Roman North Africa, but their remains are scarce and have not yet been subject to dating or biomolecular analysis which means their species assignment is unconfirmed (63, 64).

#### Extinctions and population replacements.

The native *D. mesopotamica* distribution contracted substantially through time and by the medieval period was replaced in eastern Turkey by *D. dama* (Fig. 2). The Balkan population of *D. dama* was likely extinct by the end of the medieval period (Fig. 2*C*). Of the translocated populations, the Persian fallow deer of Mallorca went extinct around the seventh century CE (31) and the population on Cyprus disappeared by the late medieval/early modern period (65). The *D. dama* population established in northern Europe during the Roman period vanished rapidly following the Empire’s withdrawal, and new populations were re-established centuries later (Fig. 2 *B* and *C*).

For Britain, our study overturns the received wisdom that fallow deer were brought from the Norman kingdom of Sicily following the Norman Conquest of 1066 (66, 67). Our skyline plot (S1 Fig. 2) suggests an introduction ~1000 CE and this model is supported by the evidence from the site of Goltho, Lincolnshire. Isotope analyses of the Goltho deer indicate they were born and raised locally (ref. 33 and Fig. 4) and direct dating suggests that a population was established before the Norman Conquest, by at least 1000 CE (Fig. 3). The possibility that these deer were derived from Sicily can be discounted from the genetic evidence which demonstrates that the North European medieval deer are unrelated to either the Roman or Western Mediterranean populations (Figs. 1 and 2*C*). Instead, they are more closely related to Anatolian deer, both of which lack a 21 bp mtDNA insertion present in 88% of modern Italian and Spanish individuals (4).

#### Elite exchange and colonial expansion.

Following the second introduction to Britain, the maintenance of fallow deer within parks became a statement of elite identity (33) and by the early 13th century, parks and fallow deer had been established in Ireland by Anglo-Norman colonial powers (32, 68, 69). About this time fallow deer were exported to France, sent by King Henry II to stock King Philip II’s park at Vincennes (70).

In fact, England was likely the source of the deer reintroduced to other countries of northern Europe. A 16th/17th-century specimen from the Boussu castle, Belgium (71) was found to have a unique haplotype (H25) closely related to the most common English haplotype (Fig. 2*C*). The 16th/17th-century specimen from Bierstraat-The Hague (Netherlands) yielded insufficient aDNA to understand its relationship to the broader dataset. Nevertheless, the oxygen isotope results suggest that it may have been a first-generation import from England (Fig. 4) and historical evidence supports this possibility. For example, the 17th century Dutch hunting manuscript, Jacht-Bedryff, notes that Maurice of Nassau (later Prince of Orange) acquired 100 fallow deer from England to stock The Hague forest (72).

The combination of historical research and genetic results indicates that England was the source of the fallow deer exported across the British Empire. The *Dama* of the Caribbean island of Barbuda (H63) is closely related to English deer, which is consistent with documentary evidence that fallow deer were transported to the island, along with many African slaves, by the Codrington family of Gloucestershire (73). The meaning attached to these Barbudan deer changed through time. Initially, they were a symbol of colonial authority and dominance, but after the slave emancipation of 1834, fallow deer became a symbol of freedom, adopted as Barbuda’s national animal. Today, fallow deer are an important part of Barbuda’s economy and cultural heritage but, as an introduced “alien” species, they fall outside legal protection. This is despite clear threats from over-hunting and natural disasters, such as hurricane Irma that devastated the island in 2017, which have put the culturally important population at risk (73, 74).

### Implications for Fallow Deer Management and Conservation.

The Barbudan fallow deer are just one of many global populations that possess cultural importance. Yet, it is precisely the close association with humans, and particularly their human-assisted translocation, that excludes them from IUCN protection. We argue that the cultural heritage represented by a species should be taken into consideration when conservation decisions are being made.

The results presented here serve as a warning about the vulnerability of island fallow deer. Ancient introductions to Crete, Chios, Cyprus, Sicily, Sardinia, Mallorca, and Roman Britain all went extinct (Fig. 2*D*). The modern Barbudan population could follow a similar trajectory without a conservation plan akin to that which allowed the Rhodes fallow deer to endure from their Neolithic introduction. It is the deer from Rhodes, along with those from Italy and Portugal, that preserve traces of the now extinct refugial population that once inhabited the Balkans.

There are several active campaigns to re-establish fallow deer in the Balkans and preserve the last remaining wild herd at Daği-Termessos National Park, Turkey. Without knowledge of the species’ deep-time biomolecular and phylogeographic history, deer are being sourced from the least appropriate populations. For instance, those being reintroduced to the Balkans possess Anatolian ancestry (Fig. 2*D*). Furthermore, these Anatolian deer are being introduced to regions that have, for thousands of years, preserved deer with Balkan ancestry (Fig. 2*D*). Whilst Anatolia-derived deer are increasing in number around the world, the Daği-Termessos herd is still under threat. Our contention is that North European deer of Anatolian ancestry could be introduced to the Daği-Termessos park, whilst Iberian/Italian/Rhodes deer populations would be a better source for Balkan rewilding projects.

## Conclusion

This study combined zooarchaeology and ancient and modern biomolecular datasets with evidence from Humanities disciplines to reveal new insights into the history of both fallow deer and the people who transported them. We argue that after the Last Glacial Maximum, Persian fallow deer were more widespread than has previously been proposed, whilst European fallow deer were likely restricted to Anatolia and the Balkans, and two distinct populations existed on either side of the Bosporus. Our integrated study suggests early translocations of deer as a viable alternative to fallow deer surviving anywhere else outside these regions.

Tracing their spread from these refugia reveals that fallow deer were repeatedly sourced from the furthest available populations: The deer on Neolithic Chios (and likely Rhodes) derived from the Balkans, rather than nearby Anatolia; those on Roman Mallorca were *Dama mesopotamica* rather than the *Dama dama* which could have been acquired from the Iberian peninsula; and the deer reintroduced to medieval Britain were brought from Anatolia instead of Iberia or Italy. This reflects the human desire to possess the exotic which, across cultures, is linked to concepts of power and otherworldliness. Not surprisingly then, the earliest translocations of fallow deer are linked to the religious cults of Artemis and Diana.

Ancient dispersals of people, ideas, and animals are widely celebrated as cultural heritage. However, the more recent the migrations, the more negative the attitudes toward them. Such perceptions can translate into animal management and policy making. For instance, the fallow deer of Rhodes were introduced during the Neolithic and are viewed as a cultural asset, protected by Greek law and featured on the IUCN Red List. The fallow deer of Barbuda are equally culturally significant as the island’s national animal, yet they have no legal protection and are labeled as “invasive” within the conservation literature. In truth, they are dismissed only because their introduction occurred too recently to have acquired a patina of age-based authenticity.

Given the planet’s biodiversity crisis, it is time to rethink our attitudes to animals. Whilst many species may legitimately be labeled as invasive, this is not true of all translocated populations and some do deserve protection. Preoccupation with native and wild species can come at the expense of (often equally endangered) translocated animals that are not only critically entangled with human history but also offer a conservation resource for replenishing diminished autochthonous populations.

## Materials and Methods

Spatiotemporal shifts in European and Persian fallow deer distribution were initially reconstructed through synthesis of the zooarchaeological literature. Reports referencing the presence of fallow deer were collated (n = 336) and the frequency of fallow deer (relative to main mammals) within each assemblage was calculated (Dataset S2). The location and frequency data were mapped for three key chronological periods—Neolithic and Bronze Age (8000 to 1200 BCE), Iron Age and Roman (1200 BCE to 500 CE), and medieval and early modern (500 to 1800 century CE)—to create Fig. 2 *A*–*C*.

To add resolution to the zooarchaeological survey, 635 osteological samples (archaeological, historical, and modern) were acquired from sites across the fallow deer’s ancient and modern range (Dataset S1). Samples were subject to full-suite analysis using the following techniques (*SI Appendix* for full details):

### Zooarchaeological Analysis.

Contextual information (site type, date, and associated archival data) was recorded for each specimen, which was identified to skeletal element and examined for evidence of taphonomic process and pathology. Metrical analysis (75) and age determinations (76) were undertaken to assist with species assignment and demographic profiling. Osteometric data were compared against those published on the Deer Bone Database https://www.nottingham.ac.uk/zooarchaeology/deer_bone/search.php.

### Isotope Analysis.

A total of 418 specimens were submitted for multi-element isotope analyses, including carbon and nitrogen (n = 418), oxygen (n = 31), strontium (n = 18), and sulfur (n = 22). Collagen extractions were undertaken at the University of Nottingham. Other preparation methods and analyses were undertaken at the National Environmental Isotope Facility (formerly NERC Isotope Geosciences Laboratory) at the British Geological Survey, Keyworth, UK. Oxygen data were compared against Miller et al.’s modern baseline (37).

### Chronologies and Radiocarbon Dating.

Dating of the archaeological specimens was based largely on contextual association. To check issues of stratigraphic migration [which have been noted in smaller animals (50)] and refine the chronology of fallow deer translocations, published radiocarbon dates were collated (n = 9) and key specimens (n = 23) were directly dated: 21 at the University of Oxford’s Radiocarbon Accelerator Unit (ORAU) UK and two at Beta Analytic (USA).

### Genetic Analysis.

The genetic data generation and analysis was carried out at the Molecular Ecology Group at the Department of Biosciences, Durham University, UK. In order to maximize both the number of variable positions and to be able to compare to data generated from modern specimens, we targeted a 532 bp fragment of the 5′ end of the control region of the mitochondrial genome using a combination of overlapping primer pairs. PCR products were sequenced using the Sanger method on an ABI 3100 automated sequencer at DBS Genomics, Durham University.

Out of 561 ancient specimens, we generated the entire fragment from 190 European fallow deer and generated a sequence alignment alongside 219 modern samples (Datasets S3 and S4), published in Baker et al. (4). For discrimination between *D. dama* and *D. mesopotamica,* we used a 128 bp sub-fragment from the same control region sequence. This allowed us to identify 38 ancient specimens as Persian fallow deer (*SI Appendix*, Fig. S1).

All sequences were aligned using the MUSCLE algorithm (77) as implemented in Geneious v. R6 (www.geneious.com, ref. 78). The relationship amongst haplotypes was examined by constructing both median-joining networks (79) in NETWORK v. 3.1.1.1 (www.fluxus-engineering.com) and a Bayesian phylogeny within MrBayes v. 3.2.6 (80). The demonstrated phylogenetic distinctiveness of the two subspecies *D. dama* and *D. mesopotamica* (31) allowed us to confirm species identifications when zooarch assessments were equivocal. This was based on 18 fixed differences out of the 128bp sequence. Additional details pertaining to the data generation, analyses, and GenBank accession details (Dataset S5) are found in *SI Appendix*.

## Supplementary Material

Appendix 01 (PDF)

Dataset S01 (XLSX)

Dataset S02 (XLSX)

Dataset S03 (XLSX)

Dataset S04 (XLSX)

Dataset S05 (XLSX)

## Data Availability

Genetics data have been deposited in GenBank (https://www.ncbi.nlm.nih.gov/nuccore (35, 36): H1 OR220344 H2 OR220345 H3 OR220346 H4 OR220347 H5 OR220348 H6 OR220349 H7 OR220350 H8 OR220351 H9 OR220352 H10 OR220353 H11 OR220354 H12 OR220355 H13 OR220356 H14 OR220357 H15 OR220358 H16 OR220359 H17 OR220360 H18 OR220361 H19 OR220362 H20 OR220363 H21 OR220364 H22 OR220365 H23 OR220366 H24 OR220367 H25 OR220368 H26 OR220369 H27 OR220370 H28 OR220371 H29 OR220372 H30 OR220373 H31 OR220374 H32 OR220375 H33 OR220376 H34 OR220377 H35 OR220378 H36 OR220379 H37 OR220380 H38 OR220381 H39 OR220382 H40 OR220383 H41 OR220384 H42 OR220385 H43 OR220386 H44 OR220387 H45 OR220388 H46 OR220389 H47 KY564399.1 1 H48 KY564400.1 2 H49 KY564402.1 4 H50 KY564415.1 17 H51 KY564405.1 7 H52 KY564406.1 8 H53 KY564408.1 10 H54 KY564409.1 11 H55 KY564410.1 12 H56 KY564411.1 13 H57 KY564416.1 18 H58 KY564418.1 20 H59 KY564417.1 19 H60 KY564413.1 15 H61 KY564414.1 16 H62 KY564420.1 22 H63 OR531442 n/a H64 OR531443 n/a H65 KY564422.1 24 H66 KY564421.1 23,25,26 H67 KY564426.1 28 H68 KY564427.1 29 H69 KY564425.1 27 H70 KY564428.1 30,32 H71 KY564432.1 34 H72 KY564431.1 33 Dama mesopotamica XIV AF291896 n/a XV JN632630 n/a XVI OR531435 n/a XVII OR531436 n/a XVIII OR531437 n/a XIX OR531438 n/a XX OR531439 n/a XXI OR531440 n/a XXII OR531441 n/a). All other data are included in the manuscript and/or supporting information.
