# Supplementary material for: The 10,000-year biocultural history of fallow deer and its implications for conservation policy
Source: Proc Natl Acad Sci U S A. 2024 Feb 12;121(8):e2310051121. doi: 10.1073/pnas.2310051121 (PMC10895352; doi:10.1073/pnas.2310051121)
Supplement: Supplementary file 1 — Appendix 01 (PDF) [file pnas.2310051121.sapp.pdf]

Supporting Information for

The 10,000-year biocultural history of fallow deer and its implications for conservation policy

Karis H Baker[1]; Holly Miller[2], Sean Doherty[3], Howard W.I. Gray[1], Julie Daujat[2], Canan Çakırlar[4], Nikolai Spassov[5], Katerina Trantalidou[6], Richard Madgwick[7], Angela L. Lamb[8], Carly Ameen[3], Levent Atici[9], Polydora Baker[10], Fiona Beglane[11], Helene Benkert[3], Robin Bendrey[12], Annelise Binois-Roman[13]; Ruth F Carden[14], Antonio Curci[15], Bea De Cupere[16], Cleia Detry[17], Erika Gál[18], Chloé Genies[19], Günther K Kunst[20], Robert Liddiard[21], Rebecca Nicholson[22], Sophia Perdikaris[23], Joris Peters[24,25], Fabienne Pigière[26], Aleksander G Pluskowski[27]; Peta Sadler[28], Sandra Sicard[29], Lena Strid[30], Jack Sudds[3], Robert Symmons[31]; Katie Tardio[32]; Alejandro Valenzuela[33], Monique van Veen[34], Sonja Vuković[35], Jaco Weinstock[36], Barbara Wilkens[37], Roger JA Wilson[38]; Jane Evans[8], A. Rus Hoelzel[1] and Naomi Sykes[3]*.

1. Department of Biosciences, Durham University, Durham, DH1 3LE, United Kingdom
2. Department of Classics and Archaeology, University of Nottingham, Nottingham, NG7 2RD, United Kingdom
3. Department of Archaeology and History, University of Exeter, Exeter, EX4 4QE, United Kingdom
4. Groningen Institute of Archaeology, University of Groningen, Groningen, 9712 ER, The Netherlands
5. Department of Paleontology, National Museum of Natural History, Bulgarian Academy of Sciences, 1000 Sofia, Bulgaria
6. Hellenic Ministry of Culture, 106 82 Athens, Greece
7. School of History, Archaeology and Religion, Cardiff University, Cardiff, CF10 3EU, United Kingdom
8. National Environmental Isotope Facility, British Geological Survey, Nottingham, NG12 5GG, United Kingdom
9. Department of Anthropology, University of Nevada, Las Vegas, Las Vegas, NV 89154, USA
10. Historic England, Portsmouth, PO4 9LD, United Kingdom
11. Centre for Environmental Research Innovation and Sustainability, School of Science, Atlantic Technological University,Sligo, F91 YW50, Ireland.
12. School of History, Classics and Archaeology, University of Edinburgh, Edinburgh, EH8 9AG, United Kingdom
13. School of Art History and Archaeology, University of Paris 1 Panthéon-Sorbonne, 75006 Paris, France
14. School of Archaeology, University College Dublin, Dublin, DO4 V1W8, Ireland
15. Department of History and Cultures, University of Bologna, 40126 Bologna, Italy
16. Royal Belgian Institute of Natural Sciences, 1000 Brussels, Belgium
17. UNIARQ - Center of Archaeology of the University of Lisbon; Department of History, School of Arts and Humanities of the University of Lisbon, Alameda da Universidade, Lisboa 1600- 214, Portugal
18. Institute of Archaeology, HUN-REN, Research Centre for the Humanities,1097 Budapest, Hungary.
19. Bureau d'études EVEHA, 37550 SAINT-AVERTIN, Tour, France
20. Vienna Institute for Archaeological Science, Research Network Human Evolution and Archaeological Sciences, University Vienna, Vienna 1190, Austria
21. School of History, University of East Anglia, Norwich Research Park, United Kingdom
22. Oxford Archaeology Ltd, Osney Mead, Oxford OX2 0ES, United Kingdom
23. School of Global Integrative Studies, University of Nebraska-Lincoln, Lincoln, NE 68588, USA.
24.Institute of Palaeoanatomy, Domestication Research and the History of Veterinary Medicine, Department of Veterinary Sciences, Ludwig Maximilian University of Munich, Munich 80539, Germany.

25. Bavarian Natural History Collections, State Collection of Palaeoanatomy Munich, Munich 80333, Germany.
26. Department of Geography, Royal Holloway, University of London, Egham TW20 0EX, UK
27. Department of Archaeology, University of Reading, Reading, RG6 6AX, United Kingdom.
28. Independent Researcher, Buckinghamshire, United Kingdom.
29. Département de la Charente, 1616917 Angouleme Cedex 9, France,
30. Department of Archaeology and Ancient History, Lund University, 223 62 Lund, Sweden
31. Fishbourne Roman Palace, Chichester, PO19 3QR, United Kingdom.
32. Department of Classics and Ancient Mediterranean Studies. Bucknell University, 1 Dent Dr. Lewisburg, PA, 17837, USA
33. Department of Historical Sciences & Art Theory, University of Balearic Islands, Spain
34. Department of Archaeology, Municipality of The Hague, 2500 DJ Den Haag, The Netherlands
35. Laboratory for Bioarchaeology, Archaeology Department, University of Belgrade, 11000 Belgrade, Serbia.
36. Department of Archaeology, University of Southampton, Southampton, SO171BJ, United Kingdom.
37. Independent Researcher, Alghero, Italy
38. Department of Ancient Mediterranean and Near Eastern Studies, Canada.

*Naomi Sykes.
Email: N.Sykes@exeter.ac.uk

This PDF file includes:

Supporting Text
Figures S1 to S7
SI References

Other supporting materials for this manuscript include the following:
Datasets – Datasets S1-5 have been attached as excel files
- Baker K.H. et al. OR220344-OR220389, Genbank, https://www.ncbi.nlm.nih.gov/nuccore, 30 June 2023
- Baker K.H. et al. OR531435-OR531443, Genbank, https://www.ncbi.nlm.nih.gov/nuccore, 8 September 2023

Website: Deer Bone Database - https://www.nottingham.ac.uk/zooarchaeology/deer_bone/

Supporting Text

1. Methods and Materials

Spatiotemporal shifts in European and Persian fallow deer distribution were initially reconstructed through synthesis of the zooarchaeological literature. Reports referencing the presence of fallow deer were collated (n = 336) and the frequency of fallow deer (relative to main mammals) within each assemblage was calculated (Dataset S1). The location and frequency data were mapped for three key chronological periods - Neolithic and Bronze Age (8000-1200 BCE), Iron Age and Roman (1200 BCE - 500 CE), medieval and early modern (500-1800 century CE) – to create Figures 2A-C.

To add resolution to the zooarchaeological survey, physical samples (n=635) were acquired from sites across the European fallow deer's ancient and modern range (Dataset S2). Samples were subject to full-suite analysis using the following techniques:

1.2 Zooarchaeological analysis

Contextual information was recorded for each specimen, which was identified to skeletal element and examined for evidence of taphonomy and pathology. Metrical analysis (following von den Driesch (1)) and age determinations (following Bowen et al. (2)) were undertaken to assist with species assignment and demographic profiling. Osteometric data are compared against those published on the Deer Bone Database https://www.nottingham.ac.uk/zooarchaeology/deer_bone/search.php and by Karastoyanova et al.(3).

1.4 Isotope analysis

A total of 418 osteological specimens were submitted for multi-element isotope analyses, including carbon and nitrogen (n= 418), oxygen (n=31), strontium (n=18) and sulfur (n=22). All analyses were undertaken at the National Environmental Isotope Facility (formerly NERC Isotope Geosciences Laboratory) at the British Geological Survey, Keyworth, UK.

For carbon and nitrogen isotope analysis, samples were collected from as many fallow deer specimens as possible from the sites and are detailed in Dataset S2. A smaller subset was selected for sulfur isotope analysis. Collagen was extracted according to a modified Longin (4) and Brown *et al.* (5) method whereby, after the gelatinisation process, samples were filtered through 8 µm EZee filters prior to freeze drying. The isotope ratios were measured using a continuous flow-elemental analyser (Flash/EA) coupled to a ThermoFinnigan Delta Plus XL via a ConFlo III interface. Collagen carbon, nitrogen and sulfur isotope ratios ($\delta^{13}C$, $\delta^{15}N$, $\delta^{34}S$) are reported in per mil (‰) relative to VPDB, AIR and VCDT standards respectively. Isotope ratios were calibrated using an in-house reference material M1360p (powdered gelatine from British Drug Houses) with expected delta values of –20.32‰ (calibrated against CH7, IAEA) and +8.12‰ (calibrated against N-1 and N-2, IAEA) for C and N respectively. $\delta^{13}C$ and $\delta^{15}N$ analyses were done in duplicate and the average standard deviation of these pairs was $\delta^{15}N = \pm 0.05$‰ and $\delta^{13}C =$

± 0.04‰. Samples fell within the acceptable range of atomic C:N values (2.9–3.6) and percent carbon (% C) and nitrogen (% N) to sufficiently reflect *in vivo* collagen values from well preserved bones (Ambrose (6)). $\delta^{34}S$ ratios were calibrated using an in-house reference material BROC-2 (powdered broccoli) with expected delta values of 11.67‰ (calibrated against S-1 and S-2, IAEA). $\delta^{34}S$ analyses were run in duplicate and the average standard deviation of the duplicates was ±0.20‰. A modern cow bone collagen (SADCOW) was used as a secondary check standard for all elements.

For strontium and oxygen isotope analysis, the enamel surface of each tooth was abraded to a depth of >100 microns using a tungsten carbide dental burr and the residue discarded. Thin enamel slices were then cut from the teeth using a flexible diamond edged rotary dental saw. Bone and dentine samples were taken by a similar approach of abrading the surface and then cutting a small slice. For strontium analysis, samples were placed in de-ionised water at 60°C for about an hour and then rinsed three times to remove and soluble contamination. Then they were cleaned ultrasonically in high purity water to remove adhering particulate material, rinsed again several times, dried and weighed into pre-cleaned Teflon beakers. The samples were then mixed with ^{84}Sr tracer solution and dissolved in Teflon distilled 8 M HNO_3. Strontium was collected using Dowex resin columns (Dickin (7)).

The Sr isotope composition and concentrations were determined by thermal ionization mass spectroscopy (TIMS) using a Thermo Triton multicollector mass spectrometer. Samples were run at c. 3V using single Re filaments loaded using TaF following the method of Birck (8). The international standard for $^{87}Sr/^{86}Sr$, NBS987, gave a value of 0.710251 ± .000005 (n=19, 2s) during the analysis of these samples. Blank values were in the region of 100pg.

For $\delta^{18}O_C$ analysis, approximately 3 mg of clean, powdered enamel was loaded into glass vials and sealed with septa. The vials were transferred to a hot block at 90°C on a Multiprep system (GV Instruments, Manchester, UK). The vials were evacuated and four drops of anhydrous phosphoric acid were added. The resultant CO_2 is collected cryogenically for 14 min and transferred to a GV IsoPrime dual inlet mass spectrometer.

The resultant isotope values were normalized to the V-PDB scale using an in-house carbonate reference material (KCM) calibrated against NBS-19 certified reference material. The $\delta^{18}O_C$ values were then converted into the V-SMOW scale using the published conversion equation of Coplen (9) (V-SMOW=1.03091 x $\delta^{18}O$ PDB + 30.91). The 1σ reproducibility of the KCM reference material for this set of analyses was calculated by analysis of variance (ANOVA), which separates the within-batch variation from the between-batch variation (Miller and Miller (10)). Oxygen data are here compared against Miller et al.'s (11) modern baseline. Average annual temperature data for the sample sites in Miller et al. 2019 were used to calculate the temperature estimates in Figure 4, which are provided as an interpretative guide.

1.5 Radiocarbon dating

To refine the chronology of fallow deer translocations, published radiocarbon dates were collated (n=9) and an additional 23 specimens were dated: 21 at the University of Oxford's Radiocarbon Accelerator Unit (ORAU) UK and 2 at Beta Analytic (USA).

The samples dated at ORAU were processed using the gelatinisation and ultrafiltration protocols described by Brock *et al.* (12) and Bronk Ramsey *et al.* (13). The samples were then combusted, graphitised and dated by Accelerator Mass Spectrometry (AMS) as described by Brock *et al.,* (12), Dee and Bronk Ramsey (14), and Bronk Ramsey *et al*. (15). ORAU maintains a continual programme of quality assurance procedures, in addition to participation in international inter-comparisons (Scott *et al.* (16)), which indicate no laboratory offsets and demonstrate the validity of the precision quoted. All samples produced results, which have been calibrated using OxCal 4.4.2 (Bronk Ramsey (17)) using IntCal20 calibration curve (Reimer *et al.* (18)).

The samples submitted to Beta Analytic were pre-treated, involving decalcification using 0.5M HCl, removal of humates using 0.1 M NaOH, then demineralisation using 0.5M HCl. The samples then followed the gelatinisation and ultrafiltration protocols described by Brock *et al.* (12) and Bronk Ramsey *et al.* (13). The samples were then combusted, graphatised and dated by Accelerator Mass Spectrometry. All samples produced results, which have been calibrated using OxCal 4.4.2 (Bronk Ramsey (17)) using IntCal20 calibration curve (Reimer *et al.* (18)).

1.6 Genetic analysis

We collected 780 samples (561 of which are ancient/osteological and 219 of which were modern/soft tissue) from across the *D. dama* species range (see Dataset SI 2 and 3). We chose mtDNA because it is haploid and matrilineal, and so tracks the movement of individuals (not distorted by sexual recombination), and for short segments can be amplified from large numbers of ancient samples. As seen from an earlier study (Baker et al. (19)), 532bp of the control region was sufficient for clear differentiation among regions, and consistent with inference from nuclear markers.

Ancient DNA extraction: Bone samples were surface sanded (to ensure removal of surface contaminating DNA) and a small segment of bone was then cut (~1cm^3) using a variable speed Dremel™ drill. Bone powder was obtained from cut segments by placing in a mikrodismembrator (Sartorius). 50mg of this bone powder was then incubated overnight at 50°C with 1 mL of extraction buffer (0.5 M EDTA at pH 8.0, 0.5% SDS and 0.5 mg/mL proteinase K) in a 1.5 mL tube. DNA was extracted using a QIAquick purification kit™ (Qiagen) and stored at -20°C.

Precautions to avoid contamination were taken during every stage of aDNA extraction and PCR set up, which took place in a separate laboratory dedicated to ancient DNA research, free from contemporary

DNA or PCR product. No laboratory materials or clothing were transferred from the post amplification rooms to the ancient laboratory. All equipment, plasticware and DNase & RNase-free deionized sterile water was autoclaved in a dedicated aDNA autoclave. All work surfaces and equipment were thoroughly cleaned with 10% bleach (sodium hypochlorite) followed by 70% ethanol. Surfaces, equipment and solutions were also routinely exposed to UV light for at least 10 minutes. All extractions and PCR work was carried out in class II PCR hoods. Negative extraction and PCR controls (1 sample in every 5) were both included to detect potential contamination in reagents and cross contamination between samples. 30% of samples were replicated by extracting twice from independent samples of the same bone.

Mitochondrial D-loop sequencing and cloning: For ancient samples a 532 base pair subsection from the 5' end of the mitochondrial control region was PCR amplified using a combination of the overlapping primers listed below. PCR Reactions (25µl) contained 2 µl of DNA extract, 1X Multiplex *PCR* Kit (Qiagen) and 0.2 µM of each primer. Thermal cycling conditions were as follows: an initial denaturation of 15 minutes at 95°C followed by 45 cycles of 30 seconds at 95°C, 90 seconds annealing, 45 seconds at 72°C followed by a final extension for 30 minutes at 60°C. Negative controls (no DNA) were used for all PCR runs. Purified PCR products were sequenced by the Sanger method on an ABI 3100 automated sequencer at DBS Genomics, Durham University. Samples showing any sequencing ambiguities were replicated for all steps.

For some samples PCR product was cloned into the pGEM-T Easy vector (Invitrogen) and transformed into JM109 competent cells (by heat-shock for 45-50 seconds). After blue/ white selection, positive colonies were miniprepped and the insert DNA amplified by PCR using T7 and Sp6 primers. A minimum of 6 clones were sequenced on an ABI automated sequencer from 6 samples chosen at random to check for potential contamination or incorrect base calls. During cloning and sequencing, no evidence of nuclear copies was detected.

Modern samples were extracted and amplified following the methods as described in Baker et al (19) (where these data are also published). For this study all sequences were trimmed to the comparative, consensus region amplified in ancient samples.

Forward and reverse primers used to amplify a partial region of the mtDNA d-loop

Primer set	Primer	Primer sequence 3'-5'	PCR product length	Annealing Temperature
Fallow 1f and 1r	Fallow 1f	TTTAAACTATTCCCTGACGCTTA	253	50.1
	Fallow 1r	AAGCATGGGGTATATGTAATGT		
Dama 4f and 5r	Dama 4f	ACATTACATTATATACCCCATGCTT	364	59
	Dama 5r	CACACAGTTATGTGTGAGCA		
**Alternative primers for 4f and 5r region**				
Ps6f and 6r	PS6F	TCTGGTTYTTTCTTCAGGGCCA	231	57.2
	PS6R	GGGATGCTCAAGATGCAGTT		
RD3F and 3r	RD3F	GATCACGAGCTTGATTACC	149	57.2
	RD3R	TTAGGTGAGATGGCCCTGAA		
2f new and 2r new	2f new	CAGCAAAACATGTGATACAAYCC	239	57.2
	2r new	GCGGCATGGTAATTAAGCTC		

Genetic networks and phylogeny: MtDNA sequences were aligned using the MUSCLE algorithm (Edgar (20)) as implemented in Geneious v. R6 (http://www.geneious.com, Kearse *et al*. (21)). The relationship amongst haplotypes was examined by constructing median-joining networks (Bandelt et al. 1999) in NETWORK v. 3.1.1.1 (www.fluxus-engineering.com).

A Bayesian phylogeny was estimated using the combined ancient-and-modern dataset (72 haplotypes) based on 594 bp of mtDNA D-loop sequence. A database sequence of Persian fallow deer (*Dama mesopotamica*) was used as an outgroup (accession: AF291896.1). MrBayes v. 3.2.6 (Huelsenbeck & Ronquist (22)) was implemented using the CIPRES Scientific Gateway v. 3.3 (Miller *et al.* (23)) to estimate Bayesian phylogenies for each dataset. Four independent Markov chains (three heated) were run over 30,000,000 MCMC generations, with a 10% burn-in, and a sampling frequency of 1,000 generations. Each analysis was run twice to confirm convergence. Various diagnostic outputs were consulted to confirm convergence had been achieved and that sufficient iterations had been implemented, particularly the Effective Sample Size (ESS) and Potential Scale Reduction Factor (PSRF) values. All ESS values were greater than 100 and all PSRF values approached one. The best model of evolution was inferred using PartitionFinder v. 1.0.1 (Lanfear *et al*. (24-25)) considering all the evolutionary models available to MrBayes. For all datasets, the HKY model (Hasegawa *et al*. (26)), with gamma distributed rate variation among sites (G), was chosen, based on the Bayesian Information Criterion (BIC) metric.

1.7 Additional Results

1.7.1 Genetics

A Bayesian phylogeny was estimated for Persian fallow deer *D. mesopotamica* sequences *n* = 51, and European fallow deer *D. dama* haplotypes (see Fig 1) using a mtDNA D-loop dataset with reduced resolution (128 bp). Database sequences of *D. mesopotamica* were included (accession: AF291896.1, AF291896, JN632630.1, NC_024819.1) and red deer (*Cervus elaphus*) was used as an outgroup (accession: AB012385). Sequences were aligned in Geneious and exported to R where they were reduced to haplotypes, *n* = 21 (European = 13; Persian = 9), using the packages *ape* (Paradis (27)) and *pegas* (Paradis, (28)). MrBayes v. 3.2.6 (Huelsenbeck & Ronquist (22)) was implemented as reported for Fig 1. The GTR substitution model was used, as identified in PartitionFinder based on the AICc metric.

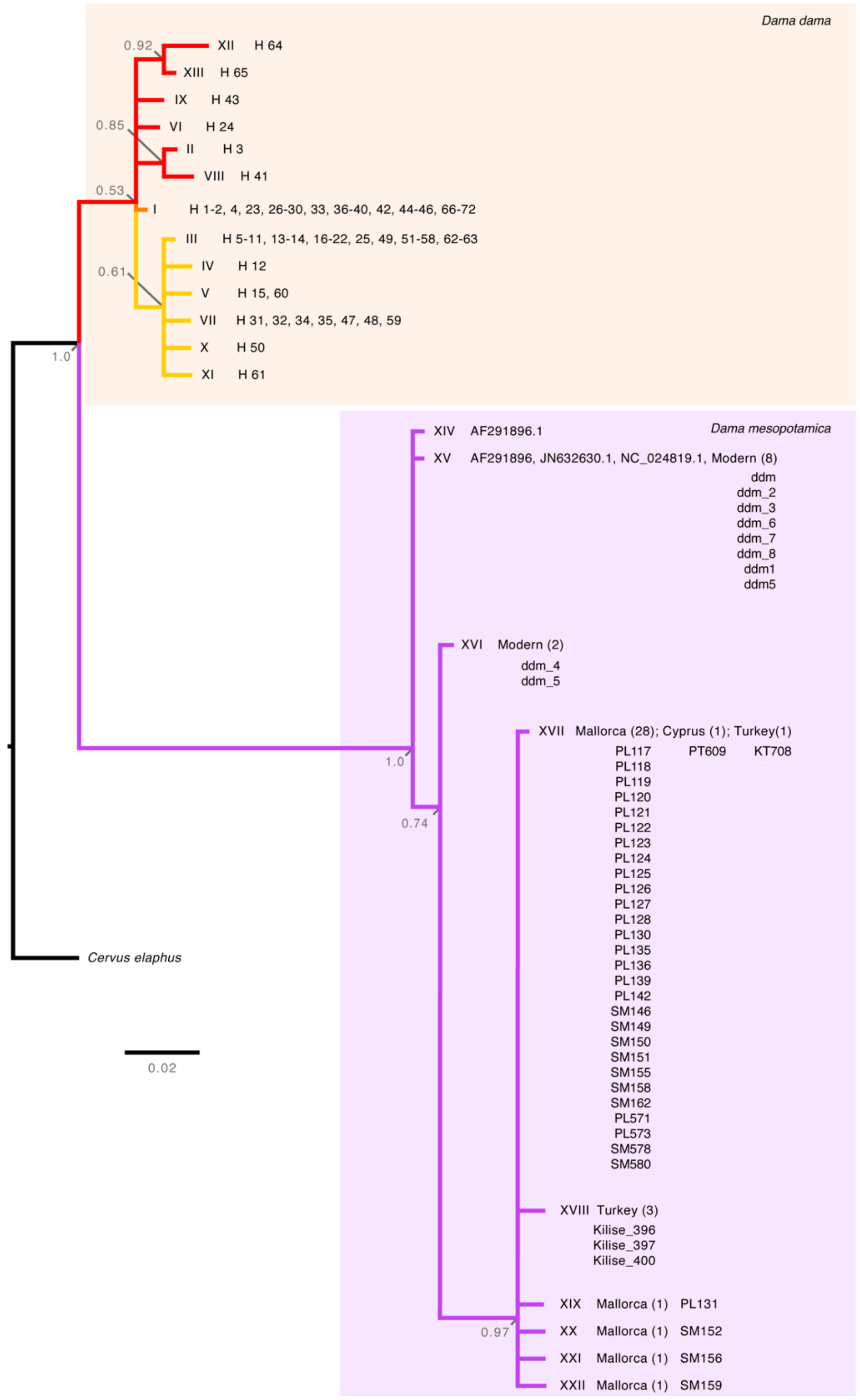

Figure S1: Bayesian phylogeny estimated using a 128 bp mtDNA D-loop to discriminate between *D. dama* and *D. mesopotamica*. Posterior probabilities given at respective nodes. Scale bar = substitutions/site**.**

Bayesian skyline plot

A subset of sequences, composed of English Roman, medieval, early modern and modern samples (listed below), were utilised in a Bayesian Skyline analysis (Ho & Shapiro (29)) as implemented in BEAST v1.8.0 (Drummond & Rambaut (30)). A lognormal uncorrelated relaxed clock and piecewise-constant skyline model with 10 groups was used. A HKY substitution model (Hasegawa *et al*. (26)), with gamma distributed rate variation among sites (G) was applied, identified based on the Bayesian Information Criterion metric as the most appropriate model considering those available to BEAST in PartitionFinder v. 1.0.1 (Lanfear *et al*. 24-25). Operators were auto-optimised. Five runs were performed, each with 20 million chains including 10% burn-in. The runs were assessed for convergence in Tracer v.1.6 (Rambaut *et al.* (31)) and combined in LogCombiner v. 1.7.5 (Drummond & Rambaut (30)), yielding 100 million chains in total (including 10% burn-in). ESS values for all parameters were above 100, most were above 200, suggesting an appropriate number of iterations had been performed. The combined log and corresponding combined trees were used to generate a Bayesian Skyline plot in Tracer.

Figure S2: Bayesian skyline plot

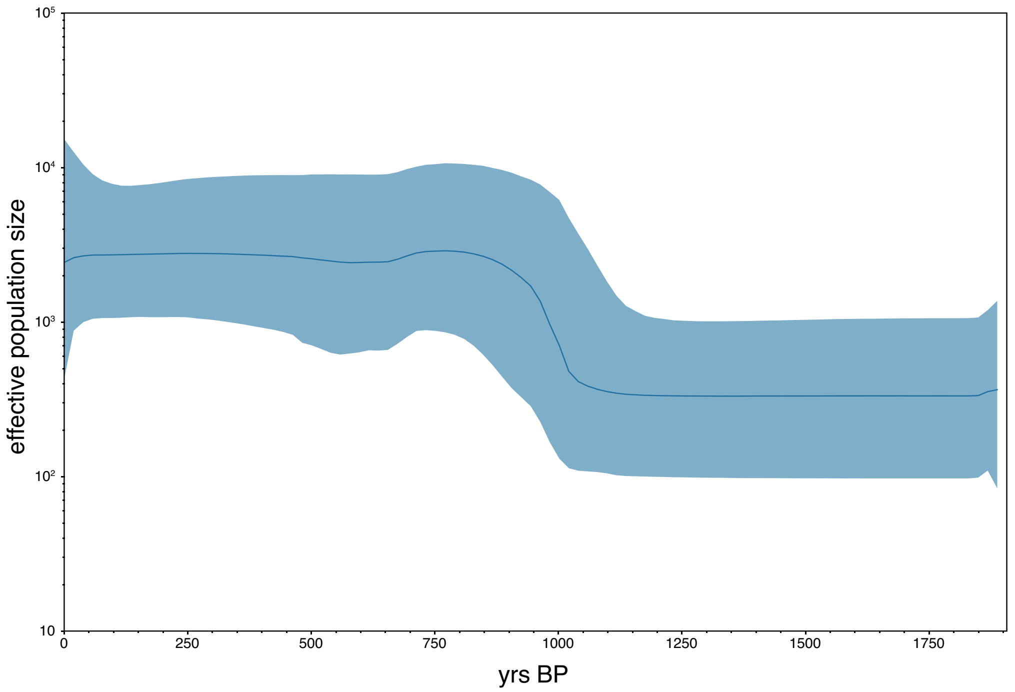

Samples utilized in the Bayesian Skyline analysis.

Date	Sample & location	Date	Sample & location	Date	Sample & location5
200	BeltonHouse_post_med_503	665	Cheddar_Palaces_427	Modern	Bradfield_wood_Suffolk
215	Hungate_york_237	697	Faccombe_Netherton_383	Modern	Ashridge_Oxford
215	Hungate_York_247	697	Faccombe_Netherton_385	Modern	Bradfield_wood_Suffolk_2
265	Hungate_York_239	697	2_Faccombe_Netherton_391	Modern	Dartmoor
315	Hungate_York_251	697	Faccombe_Netherton_392	Modern	Dartmoor_2
425	Dudley_Castle_563	697	Faccombe_393	Modern	Dartmoor_3
465	Hungate_York_232	697	Faccombe_Netherton_394	Modern	Dartmoor_4
465	Hungate_York_235	724	Dudley_Castle_515	Modern	Dartmoor_5
465	Hungate_York_236	765	Lewes_Castle_504	Modern	Dartmoor_6
465	Hungate_York_238	770	Carisbrooke_607	Modern	Dartmoor_7
465	Hungate_York_240	812	Binchester_194	Modern	Dartmoor_8
465	Hungate_York_245	834	Carisbrooke_604	Modern	Chilterns
465	Hungate_York_246	834	Carisbrooke_605	Modern	Chilterns_2
465	Hungate_York_249	866	Carisbrooke_606	Modern	Chilterns_3
472	Sparsholt_Villa_206	875	Faccombe_Netherton_388	Modern	Chilterns_4
472	Sparsholt_Villa_207	897	Carisbrooke_505_603	Modern	Kilverstone
472	Sparsholt_Villa_208	915	Faccombe_Netherton_386	Modern	Belton_House
472	Sparsholt_Villa_209	915	2_Faccombe_Netherton_387	Modern	belton_house
472	Sparsholt_Villa_211	930	Dudley_Castle_517	Modern	Holkham
472	Sparsholt_Villa_212	944	Dudley_Castle_561	Modern	epping_forest_Essex
472	Sparsholt_Villa_213	944	Dudley_castle_562	Modern	epping_forest_Essex_2
472	Sparsholt_Villa_214	1003	Goltho_10	Modern	epping_forest_Essex_3
472	Sparsholt_Villa_215	1003	Goltho_11	Modern	Stetchworth_Cambridgeshire
472	Sparsholt_Villa_216	1003	Goltho_12	Modern	Linken_Holt
472	Sparsholt_Villa_217	1003	Goltho_13	Modern	Houghton
493	Dudley_Castle_565	1003	Goltho_14	Modern	Houghton_2
493	Dudley_Castle_566	1003	Goltho_15	Modern	attingham_deer_park
493	Dudley_Castle_569	1003	Goltho_17	Modern	belton_house_2
493	Dudley_Castle_570	1003	Goltho_116_254	Modern	belton_house_3
500	Hyde_Abbey_200	1838	Monkton_3	Modern	Hatfield_Forest_Essex
550	Dudley_Castle_520	1841	Monkton_6	Modern	Hatfield_Forest_Essex_2
550	3_Dudley_Castle_522	1843	Monkton_5	Modern	Lyme_Park_Cheshire
550	4_Dudley_Castle_523	1851	Monkton_1	Modern	revesby_Lincolnshire_UK
565	Hungate_York_231	1864	Monkton_2	Modern	belton_house_4
591	Faccombe_Netherton_384	1865	Monkton_East_Kent_114	Modern	attinhgham_deer_park
600	Arcadia_Buildings_202	1865	Monkton_Tothill_Street_203	Modern	revesby_Lincolnshire_UK_2
640	Eynsham_Abbey_255	1865	Monkton_Tothill_street_205	Modern	Hatfield_Forest_Essex_3
640	Eynsham_Abbey_254	1865	Fishbourne_78	Modern	Holkham_2
656	Dudley_Castle_514	1865	Fishbourne_80	Modern	revesby_Lincolnshire_UK_3
656	Dudley_Castle_519	1865	Fishbourne_81	Modern	scivelesby_Lincolnshire_UK
656	Dudley_Castle_521	1865	Fishbourne_83	Modern	scivelesby_Lincolnshire_UK_2
656	Dudley_Castle_567	1865	Fishbourne_90	Modern	lockinge_Oxfordshire
656	Dudley_Castle_568	1865	Fishbourne_91	Modern	lockinge_Oxfordshire_2
664	Hungate_York_244	1865	Fishbourne_98	Modern	lockinge_Oxfoordshire

665	Hungate_York_233	1865	Fishbourne_99	Modern	Stechworth_Cambridgeshire
665	Hungate_York_234	1865	Fishbourne_103	Modern	Holkham_3
665	Hungate_York_241	1865	Fishbourne_102	Modern	horringer
665	Hungate_York_248	1865	Fishbourne_105	Modern	Whipsnade_zoo
665	Hungate_York_250	1865	Fishbourne_111	Modern	Houghton_3
665	Cheddar_Palaces_222	1865	Fishbourne_115	Modern	Wytham
665	Cheddar_Palaces_223	1907	Monkton_7	Modern	scivelesby_Lincolnshire_UK_3
665	Cheddar_palaces_224	1907	Monkton_8	Modern	horringer_2
				Modern	Bradfield_Wood_Suffolk
				Modern	Lockinge
				Modern	Lockinge_2
				Modern	Holkham_4

1.7.2 Osteometric analysis

To examine the morphological differences between populations from Anatolia and the Balkans, five linear metrics ("GLl", "GLm", "Bd", "Dl", "Dm") were analysed from the astragalus (Anatolian n = 34 and Balkan n =56) collected following protocols established by Von Den Driesch (1).

Differences between geographical groups were compared across all five metrics using a pairwise comparison Wilcoxon rank sum test with resulting p-values adjusted for multiple comparisons using the Bonferroni correction (Figure S3). Results indicate that there are significant differences in the individual metrics between Anatolian and Balkan specimens across four of the five metrics, with no differences observed in Dm (p=0.067).

A principal component analysis was performed on the combination of all five metrics, to maximise the variation between groups (Fig S4). Results of the manova on the principal component scores indicated that overall there were significant differences between the shape of the tali between Anatolian and Balkans specimens ($p \leq 4.324e-08$). The above analyses were performed in R (Version 1.3.1073) (R Core Team (32)).

Metrical analysis was also used to investigate claims that fallow deer were present on Bronze Age Sicily, at the site of Morgantina (Fig S5). The distal tibia measurement published by Bartosiewicz (33) was plotted against deer measurements from other Sicilian sites in our dataset - Campanaio and Castagna. The resulting tri-modal distribution equates to three groups identified zooarchaeologically (and in the case of specimen MO447 genetically) as red deer, fallow deer and roe deer. The Morgantina specimen is most consistent with red deer from the island.

Figure S3: Fallow deer astragalus metrics by region. Results correspond to the probability (p) of differences in mean as calculated with pairwise comparisons using a Wilcoxon rank sum test. P-values were adjusted for multiple comparisons using the Bonferroni correction. Significant results (p≤0.01) are indicated by an asterix* and are shown above the paired groups for each individual metric.

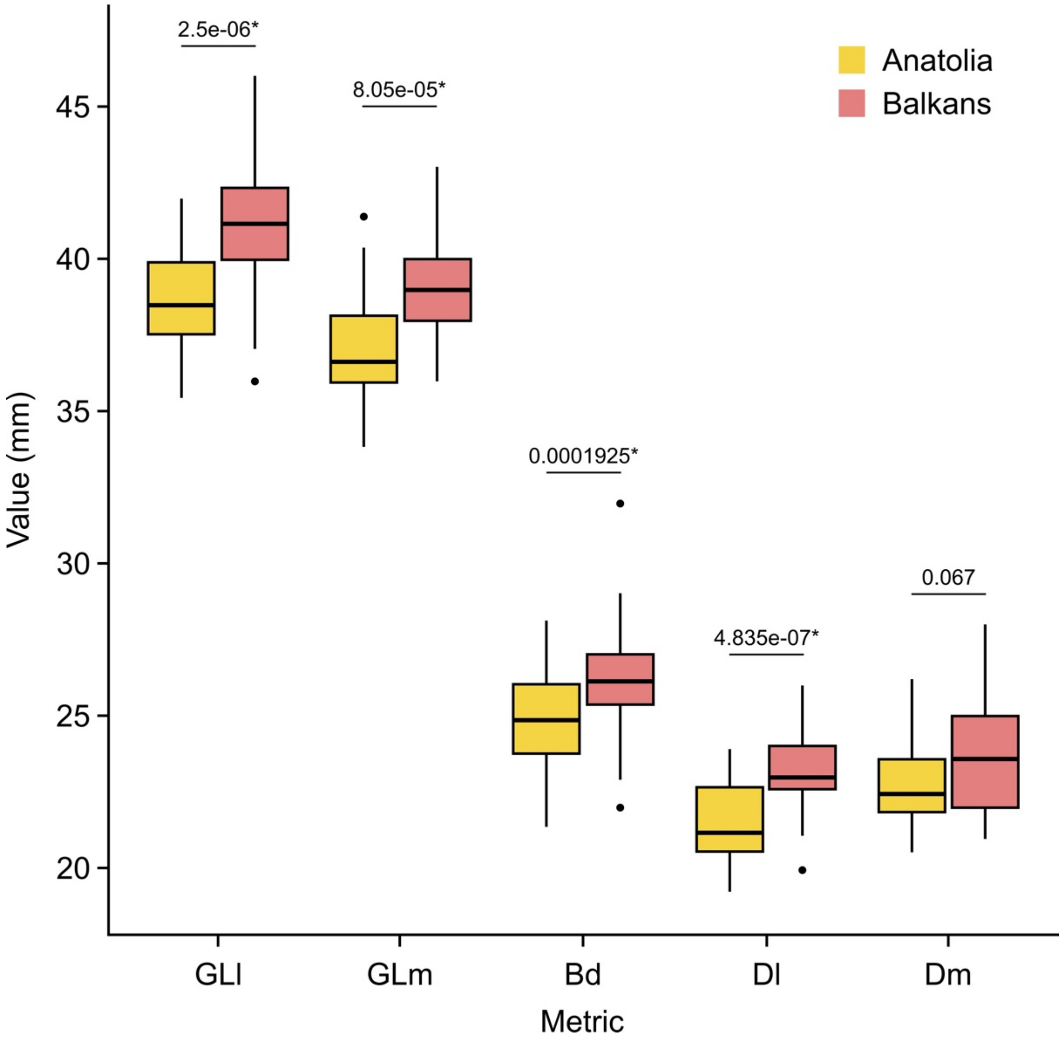

Figure S4: Visualisation of the PCA representing the pattern of astragalus shape divergence among fallow deer specimens from Anatolia and the Balkans along PC1 (x axis) and PC2 (y axis).

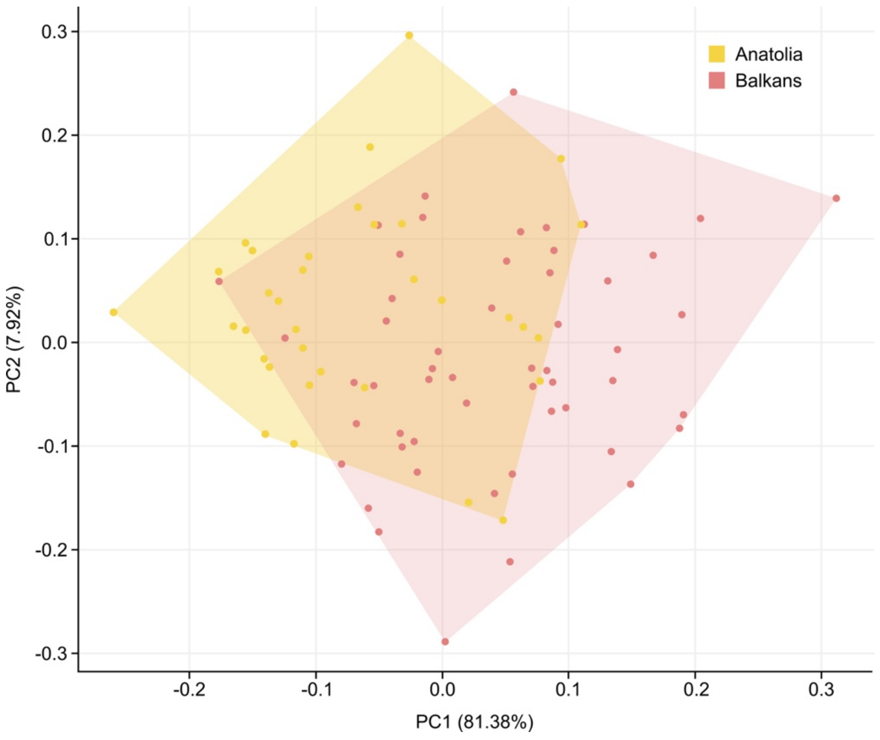

Figure S5. Distal Tibia measurements for the Bronze Age specimen from Morgantina, Sicily, shown against deer from the Roman and medieval Sicilian sites of Campanaio and Castagna.

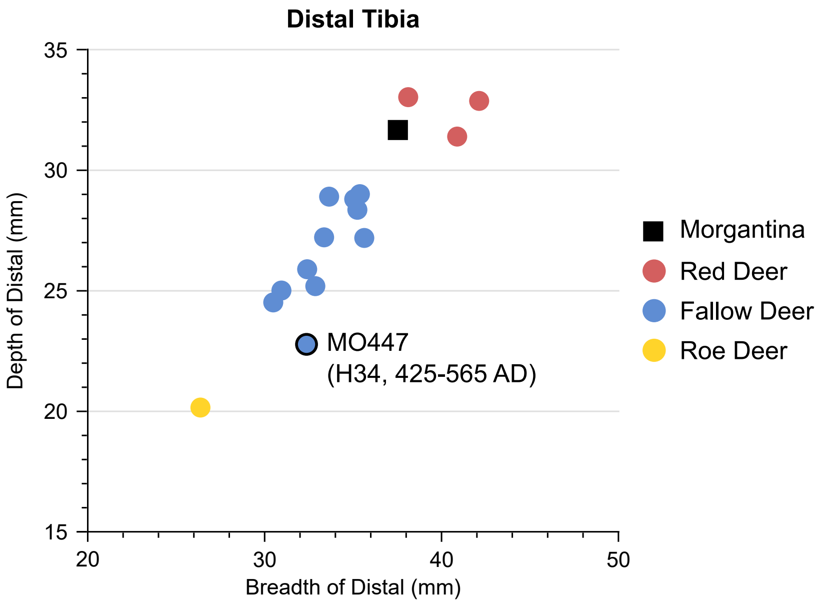

1.7.3 Isotope Analysis

Our large carbon and nitrogen isotope dataset provided the opportunity to explore spatio-temporal variations in fallow deer diet. Whilst the data for Anatolian, Balkan and Persian fallow deer demonstrated no significant differences (Fig S6) geographical partitioning of the data was apparent, with deer from the Mediterranean having higher carbon and lower nitrogen values than the deer from northern Europe.

Figure S6: Boxplot of **A)** $\delta^{13}C$ and **B)** $\delta^{15}N$ values from Anatolian, Balkan and Persian fallow deer across the Neolithic/Bronze Age, Iron Age/Roman and Medieval Periods. Number of samples for each sub-species and period in parentheses. Central line denotes the median value, the shaded box represents the inter-quartile range (IQR), the whiskers represent the values within 1.5×IQR of the upper or lower quartiles, and outliers are plotted as points.

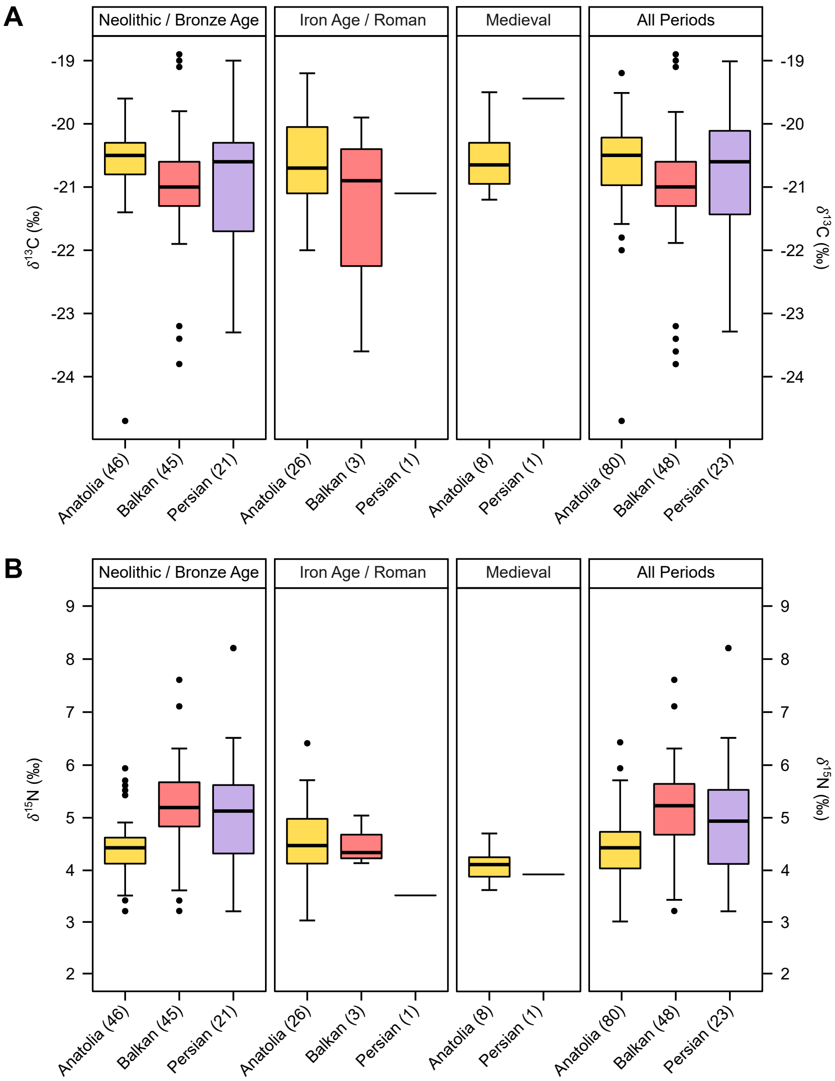

Figure S7: Geographical and temporal variation in $\delta^{13}C$ and $\delta^{15}N$ values in fallow deer. Central point denotes group mean and error bars the range. Number of samples in parentheses.

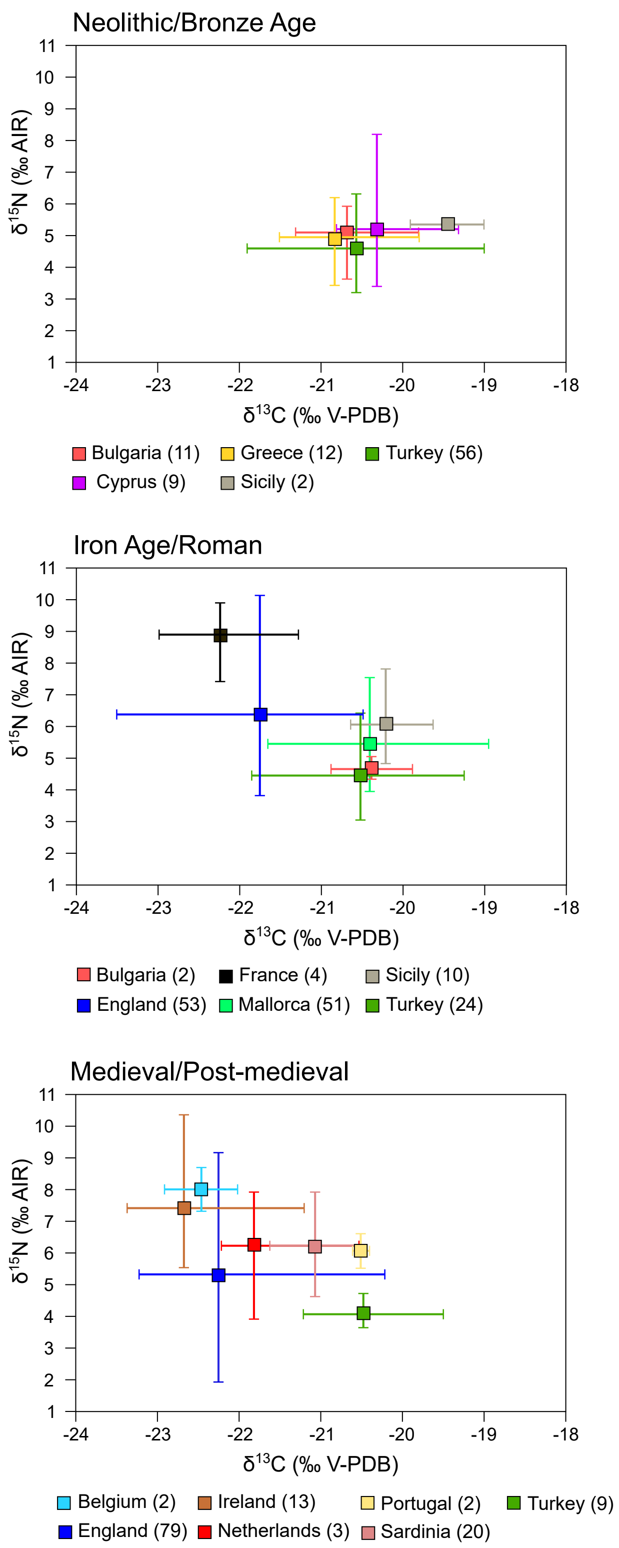

References

1. von den Driesch, A. 1976. *A guide to the measurement of animal bones from archaeological sites. Vol. 1.* Harvard: Peabody Museum Press.
2. Bowen, F., Carden, R. F., Daujat, J., Grouard, S., Miller, H., Perdikaris, S., & Sykes, N. 2016. Dama dentition: A new tooth eruption and wear method for assessing the age of fallow deer (*Dama dama*), *International Journal of Osteoarchaeology* 26(6): 1089-1098.
3. N. Karastoyanova, J. Gorczyk and N. Spassov, The natural history of the fallow deer, Dama dama (Linnaeus, 1758) in Bulgaria in prehistory and new evidence for the existence of an autochthonous Holocene population in the Balkans. *Int. J. Osteoarchaeol.* **30**, 616–628 (2020).
4. Longin, R., 1971. New method of collagen extraction for radiocarbon dating. *Nature* 230(5291): 241-242.
5. Brown, T.A., Nelson, D.E., Vogel, J.S. and Southon, J.R. 1988. Improved collagen extraction by modified Longin method. *Radiocarbon* 30(2): 171-177.
6. Ambrose, S.H., 1990. Preparation and characterization of bone and tooth collagen for isotopic analysis. *Journal of archaeological science*, *17*(4), pp.431-451.
7. Dickin, A.P. 1995. *Radiogenic Isotope Geology,* Cambridge University Press.
8. Birck, J.L., 1986. Precision K, Rb, Sr isotopic analysis: application to Rb Sr chronology. *Chemical geology*, *56*(1-2), pp.73-83.
9. Coplen, T.B., 1988. Normalization of oxygen and hydrogen isotope data. *Chemical Geology: Isotope Geoscience Section*, *72*(4), pp.293-297.
10. Miller, J.C. and Miller, J.N. 1988. Basic Statistical Methods for Analytical Chemistry. Part I. Statistics of Repeated Measurements. A Review, *Analyst* 113(9): 1351–56.
11. Miller, H. Chenery, C., Lamb, A.L., Sloane, H., Carden, R.F., Atici, L. and Sykes, N. 2019. The Relationship between the Phosphate and Structural Carbonate Fractionation of Fallow Deer Bioapatite in Tooth Enamel, *Rapid Communications in Mass Spectrometr*y 33(2): 151–64.
12. Brock, F., Higham, T., Ditchfield, P., and Ramsey, C.B. 2010. Current pretreatment methods for AMS radiocarbon dating at the Oxford Radiocarbon Accelerator Unit (ORAU). *Radiocarbon* 52(1), 103-112.
13. Bronk Ramsey, C., Ditchfield, P., and Humm, M. 2004. Using a gas ion source for radiocarbon AMS and GC–AMS, *Radiocarbon* 46(1), 25–32.
14. Dee M, Bronk Ramsey, C. (2000) Refinement of graphitetarget production at ORAU. *Nuclear Instruments and Methods in Physics Research B* 172(1–4):449–53.
15. Bronk Ramsey, C., Higham, T., Bowles, A., and Hedges, R. 2004. Improvements to the pretreatment of bone at Oxford, *Radiocarbon* 46(1), 155–63.
16. Scott, E. M., Cook, G. T., and Naysmith, P. 2010. The Fifth International Radiocarbon Intercomparison (VIRI): an assessment of laboratory performance in stage 3. *Radiocarbon*, *52*(3), 859-865.
17. Bronk Ramsey, C. 2009. Bayesian analysis of radiocarbon dates, *Radiocarbon* 51(1), 337-360.
18. Reimer, P. J., Austin, W. E., Bard, E., Bayliss, A., Blackwell, P. G., Ramsey, C. B., ... & Talamo, S. (2020). The IntCal20 Northern Hemisphere radiocarbon age calibration curve (0–55 cal kBP). *Radiocarbon* 62(4), 725-757.
19. K. H. Baker, *et al.*, Strong population structure in a species manipulated by humans since the Neolithic: the European fallow deer (Dama dama dama). *Heredity* **119**, 16–26 (2017).
20. Edgar, R.C., 2004. MUSCLE: multiple sequence alignment with high accuracy and high throughput. *Nucleic acids research*, *32*(5), pp.1792-1797.
21. Kearse, M., Moir, R., Wilson, A., Stones-Havas, S., Cheung, M., Sturrock, S., Buxton, S., Cooper, A., Markowitz, S., Duran, C. and Thierer, T., 2012. Geneious Basic: an integrated and extendable desktop software platform for the organization and analysis of sequence data. *Bioinformatics*, *28*(12), pp.1647-1649.

22. Ronquist, F. and Huelsenbeck, J.P., 2001. MRBAYES: Bayesian inference of phylogenetic trees. *Bioinformatics*, *17*(8), pp.754-755.
23. Miller, M.A., Pfeiffer, W. and Schwartz, T., 2010, November. Creating the CIPRES Science Gateway for inference of large phylogenetic trees. In *2010 gateway computing environments workshop (GCE)* (pp. 1-8). Ieee.
24. Lanfear, R., Calcott, B., Ho, S.Y.W., Guindon, S., 2012. PartitionFinder: combined selection of partitioning schemes and substitution models for phylogenetic analyses. *Mol. Biol. Evol.* **29**, 1695–1701.
25. Lanfear, R., Calcott, B., Kainer, D., Mayer, C., Stamatakis, A., 2014. Selecting optimal partitioning schemes for phylogenomic datasets. *BMC Evol. Biol.* **14**, 82.
26. Hasegawa, M., Kishino, H. and Yano, T.A., 1985. Dating of the human-ape splitting by a molecular clock of mitochondrial DNA. *Journal of molecular evolution*, *22*, pp.160-174.
27. Paradis, E. and Schliep, K., 2019. ape 5.0: an environment for modern phylogenetics and evolutionary analyses in R. *Bioinformatics*, *35*(3), pp.526-528.
28. Paradis, E., 2010. pegas: an R package for population genetics with an integrated–modular approach. *Bioinformatics*, *26*(3), pp.419-420.
29. Ho, S. Y. and Shapiro, B. 2011. Skyline-plot methods for estimating demographic history from nucleotide sequences, *Molecular Ecology Resources* 11(3):, 423-434.
30. Drummond, A.J. and Rambaut, A. 2007. BEAST: Bayesian evolutionary analysis by sampling trees, *BMC Evolutionary Biology* 7, 214.
31. Rambaut, A., Suchard, M. and Drummond, A., 2014. MCMC trace analysis tool: Tracer v. 1.6. 0.
32. R Core Team, 2018. R: A Language and Environment for Statistical Computing, Vienna, Austria. Available at: https://www.R-project.org/.
33. Bartosiewicz, L. 2012. Faunal remains (part 2, section 17), In Leighton, R. (ed), *The Archaeology of Houses at Morgantina, Sicily: Excavations of Later Prehistoric Contexts on the Cittadella (1989-2004).* London: Accordia Research Institute, University of London.
